# Not So Dead Genes—Retrocopies as Regulators of Their Disease-Related Progenitors and Hosts

**DOI:** 10.3390/cells10040912

**Published:** 2021-04-15

**Authors:** Joanna Ciomborowska-Basheer, Klaudia Staszak, Magdalena Regina Kubiak, Izabela Makałowska

**Affiliations:** Institute of Human Biology and Evolution, Faculty of Biology, Adam Mickiewicz University Poznań, 61-614 Poznań, Poland; joannac@amu.edu.pl (J.C.-B.); klaudia.staszak@amu.edu.pl (K.S.); magdalena.kubiak@amu.edu.pl (M.R.K.)

**Keywords:** retrocopies, retroposition, lncRNA, disease, parental gene, host gene, regulation

## Abstract

Retroposition is RNA-based gene duplication leading to the creation of single exon nonfunctional copies. Nevertheless, over time, many of these duplicates acquire transcriptional capabilities. In human in most cases, these so-called retrogenes do not code for proteins but function as regulatory long noncoding RNAs (lncRNAs). The mechanisms by which they can regulate other genes include microRNA sponging, modulation of alternative splicing, epigenetic regulation and competition for stabilizing factors, among others. Here, we summarize recent findings related to lncRNAs originating from retrocopies that are involved in human diseases such as cancer and neurodegenerative, mental or cardiovascular disorders. Special attention is given to retrocopies that regulate their progenitors or host genes. Presented evidence from the literature and our bioinformatics analyses demonstrates that these retrocopies, often described as unimportant pseudogenes, are significant players in the cell’s molecular machinery.

## 1. Introduction

**Figure 1:**
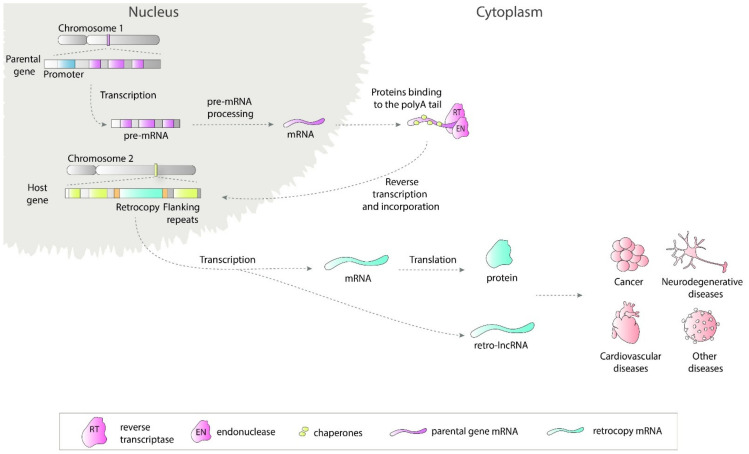
Retrotransposition of protein coding genes. The parental gene is transcribed and transported to the cytoplasm where LINE1-derived proteins bind to it. This complex is transported back to the nucleus and anneals to the broken DNA ends. Next, the reverse transcription process takes place and cDNA is inserted in the genome along with short flanking repeats. Transcription of created retrocopy can results in coding or non-coding RNA. Transcripts of retroposition-derived genes may be involved in pathogenesis of many human diseases.

Retrosequences, previously described as meaningless and biologically unimportant elements, are now recognized as evolutionarily significant, and their roles in shaping genomes, transcriptomes and proteomes have become increasingly evident [1,2,3]. This type of RNA-based gene duplicate is created through retroposition, which, together with DNA-based duplication, is known to be one of the major sources of new genes [2,4,5]. Formation of a retrocopy starts with transcription of the multiexonic parental gene (Figure 1). The mature mRNA is transported to the cytoplasm where in mammals proteins from LINE1 (Long interspersed nuclear elements 1), i.e., reverse transcriptase and endonuclease, accompanied by chaperones bind to the polyA tail. This complex is transported back to the nucleus where it anneals to the broken DNA ends and undergoes reverse transcription. Created cDNA is incorporated into new genomic surroundings. The final step includes creating short flanking repeats at insertion site, so called target site duplication (TDS). The presence of the 3′ polyA tail, and flanking sequences constitute signature of LINE-mediated retrotransposition [6,7]. These copies are regarded as “dead on arrival” pseudo(retro)genes, which usually lack introns, core promoters and other regulatory elements. Retrocopies are highly represented in placental mammals, especially primates [8]. In other genomes, *Drosophila* for example, the number of retroposed genes is relatively low [9,10]. In early studies of duplicated genes evolution, it was postulated that usually one of the duplicates accumulates mutations and becomes nonfunctional [11,12]. However, it occurred that “relaxed” selection and evolutionary freedom, which are characteristic of the majority of duplicates, may lead not only to pseudogenization but also to the acquisition of new functions [13,14]. Over time, two new phenomena related to functional evolution after duplication have been described: (i) neofunctionalization, where one copy acquires a new function and the other one keeps the original one [15], and (ii) subfunctionalization, where maintained function is shared between duplicated genes [16,17]. Additionally, as our and other studies showed, it is also possible that the retrogene (functional retrocopy) replaces its progenitor [18,19]. In the case of retrocopies, the first step needs to be obtaining regulatory elements, and there is growing evidence that many retrocopies gained the capability to be expressed over time [4,20,21].

Regardless of being described as “junk DNA” for a long time, there are numerous examples demonstrating that retrocopies may successfully work as regulatory sequences as well as crucial protein coding genes [22,23,24]. A spectacular example of retrocopy function is the *TP53* gene, a well-known tumor suppressor, and its retrocopies in elephants. Elephants have a lower-than-expected rate of cancer. It has been proposed that multiple functional retrocopies of *TP53* are involved in an increased apoptotic response by compensating for the function of their progenitor [25,26]. This compensation mechanism, in turn, might underlie the cancer resistance observed in these animals. Nevertheless, in human protein coding is relatively rare among retrogenes. For example, in RetrogeneDB2 only 106 retrocopies, out of 4611, were identified as known protein coding genes, and only 847 (18%) has intact ORF (Open Reading Frame) inherited from parental gene. Interestingly, it is quite opposite in *Drosophila* where out of 83 identified in RetrogeneDB retrocopies, as many as 81 are annotated as known protein coding genes [27]. It was found that 256 retrocopies overlaps in the human genome with annotated lncRNAs and additional 230 may act as competing endogenous RNA since they share microRNA (miRNA) targets and have correlated expression with transcripts of 232 protein-coding genes [3]. Accumulating evidence suggests that substantial number of transcriptionally active retrocopies in human act as long noncoding RNAs (lncRNAs) [14,28]. Due to their high sequence similarity, they have a natural ability to regulate, via various mechanisms, their parental genes. Additionally, since almost 40% of retrocopies are located in introns of other genes, they possess great potential to control, as antisense transcripts, their host genes.

**Figure 2:**
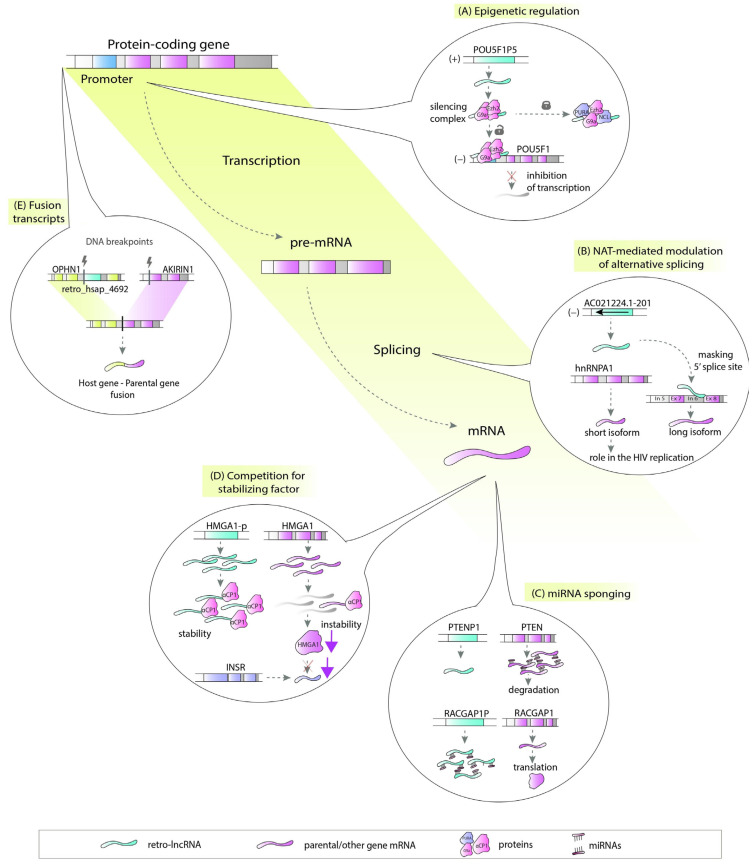
Examples of functions of human disease-related retrocopies. (**A**) RNA-mediated epigenetic regulation. *POU5F1P5* along with G9a and Ezh2 proteins create silencing complex that inhibits transcription of *POU5F1.* The complex can become blocked when proteins PURA and NCL bind to the *POU5F1P5.* (**B**) Splicing regulation. Antisense transcript of retrocopy *AC021224.1-201* can bind to the parental gene *hnRNPA1* and mask the 5′ splice site in the sixth intron. (**C**) Sponging miRNA. Under cancer condition, decreased expression level of retrocopy *PTENP1* contributes to increased miRNA binding to the *PTEN* and drives the suppressor gene on the degradation pathway. In turn, binding miRNAs to the highly expressed *RACGAP1P* allows for expression of oncogene *RACGAP1.* (**D**) Competition for stabilizing factors. Elevated expression of *HMGA1-p* (*HMGA1P8*) results in destabilization of parental gene mRNA by effective competition for a *trans*-acting cytoplasmic protein critical to mRNA stability. Low expression level of *HMGA1* gene contributes to decreased expression of the *INSR* gene which consequently manifests itself in insulin resistance. (**E**) Fusion transcripts. High sequence similarity between *AKIRIN1* and its retrocopy *retro_hsap_4692*, nested in the host gene *OPHN1* may lead to non-allelic recombination and fusion transcript formed by *AKIRIN1* and *OPHN1*.

There are a number of ways in which retrocopies may regulate their progenitors or hosts. Retrocopies can be transcribed from the antisense strand and act as natural antisense transcripts (NATs) [29]. These NATs could be involved in multiple molecular processes, including epigenetic regulation (Figure 2A), chromatin remodeling [30], or, by forming RNA:RNA duplexes, stability control, RNA editing and processing (Figure 2B) [31]. Many retrocopies work as competing endogenous RNAs (ceRNAs), also known as microRNA sponges (Figure 2C) [15,32], while others can be a source of small RNAs [33]. Retrocopies can also compete with parental genes for other molecules, such as stabilizing factors (Figure 2D) [34] or translational machinery [35]. They may also influence the splicing of the host gene as potential factors that facilitate transcriptional interference [3,36,37,38]. The impact of retrocopies on the DNA level is also noticeable since they may be involved in nonallelic homologous recombination, resulting in the formation of chimeric transcripts (Figure 2E) [3].

In light of the variety of possible functions, lncRNAs originating from retrocopies (retro-lncRNAs) can play a significant role in the cell regulatory machinery. This is especially important when their progenitors or host genes are critical in disease pathogenesis. In this review, we focus on such examples in human disorders, considering possible mechanisms of retro-lncRNA action.

## 2. Retro-lncRNAs in Cancer

Cancer constitutes a heterogeneous phenomenon with varied forms and a multifactorial basis. The role of retrocopies’ involvement in cancer has been described numerous times, and most of these retrocopies act as lncRNAs [14]. They are multifunctional, showing both oncogenic and suppressor effects [39]. In the cancer literature, we can find retro-lncRNAs that derive from parental genes referred to as “drivers”. Genetic changes within their sequences, such as mutations, give a selective growth advantage for cancer cells and thus drive cancer development [40]. Such an example is the *HMGA1* gene, which can act as a driver in liver carcinogenesis [41]. Retrocopies with no coding potential, *HMGA1P6* and *HMGA1P7*, have been indicated to be upregulated in endometrial cancer [42], ovarian cancer and thyroid cancer [43]. These additional copies can act as decoys for common microRNAs and thus regulate the expression of the parental *HMGA1* gene [43]. Another example constitutes a well-known *KRAS* gene showing driver traits in pancreatic cancer [44] and lung cancer [45], among others. Its retrocopy, *KRASP1*, which has been found to be highly expressed in prostate cancer, most likely regulates parental gene expression by sequestering microRNA [15].

Examples of retrogene-derived lncRNAs that arose from the parental gene with oncogenic function are *POU5F1P4* (*OCT4-pg4*) and *POU5F1P5* (*OCT4-pg5*), noncoding copies of the *POU5F1* (*OCT4*) gene. The *POU5F1* gene has been correlated with the occurrence of cancer stem cell populations, cell fractions increasing the risk of metastasis and recurrence in colorectal cancer samples [46,47]. *POU5F1P4* and *POU5F1P5* have been shown to be involved in the pathogenesis of hepatocellular carcinoma [48] and endometrial carcinoma [49], respectively. Most likely, these retro-lncRNAs act as microRNA sponges [48,49]. Furthermore, a regulatory mechanism based on antisense RNA-mediated epigenetic silencing of parental gene transcription has also been proposed. *OCT4-pg5*, together with other factors including G9a and Ezh2, create a silencing complex that inhibits parental gene transcription. Transcriptional inhibition could be blocked when antisense RNA is bound and sequestered by proteins such as PURA and NCL (Figure 2A) [50,51]. Another example of an oncogene-derived retro-lncRNA is *SUMO1P3*. Its increased expression has been associated with tumor size, lymphatic metastasis, differentiation and invasion in gastric cancer patients [52]. In gastric cancer, silencing of parental *SUMO1* resulted in inhibited proliferation and supported apoptosis [53]. Functional analysis showed the potential role of *SUMO1P3* in microRNA sponging and *cis*-NAT regulation of its host gene *COPA* [3].

Correlation between the expression level of lncRNA *RACGAP1P* and the promotion of early hepatocellular carcinoma recurrence [54] or breast cancer progression [55] has also been described. Moreover, the parental gene *RACGAP1* has been correlated with an aggressive phenotype in multiple cancers, including breast cancer [56] and ovarian cancer [57]. The potential mechanism is explained by *RACGAP1P* sponging of miR-15-5p (Figure 2C) [54]. Another example of a retrocopy associated with cancer is *ANXA2P2*. Cell culture studies have indicated that the *ANXA2* gene promotes the invasion of breast cancer cells [58], and elevated expression of its copy, *ANXA2P2*, has been related to an aggressive phenotype in the progression of hepatocellular carcinoma [59]. In turn, upregulation of *UBE2CP3* has been highlighted as linked to the induction of the epithelial-mesenchymal transition and thus metastasis promotion in hepatocellular carcinoma [60]. Their mechanisms of action remain unclear.

In the literature, there are also examples of retro-lncRNAs that arise from reverse transcription of suppressor genes. In examples described below, all lncRNAs mirror their parental genes and exert suppressor effects. The long noncoding RNA *INTS6P1*, along with its parental gene *INTS6*, have been termed cancer suppressors in hepatocellular carcinoma. The mechanism of their action has been connected with competition for oncogenic miR-17-5p [61]. Low expression levels of *PTENP1* are related to the cancer phenotype, and overexpression of this retro-lncRNA has been demonstrated to inhibit cancer cell proliferation [62]. Interestingly, this retrocopy is transcribed in sense as well as in antisense orientation. Under normal condition the sense transcript of *PTENP1* protects the parental gene from microRNA binding and therefore from translation inhibition. In cancer cells, down-regulation of *PTENP1* expression leads to miRNA-driven degradation of *PTEN* (Figure 2C) [15,63]. The antisense isoform of *PTENP1* may play a role as an epigenetic regulator by binding to the *PTEN* promoter and modulating its transcription [30]. Finally, downregulation of *TUSC2P1* along with its parental gene, *TUSC2,* has been correlated with the promotion of apoptosis in cancer cells, which confirms their suppressor activity [64].

In addition to these published examples, our recent analysis of RNA-seq data has shown that some cancer cell lines, including hepatocyte carcinoma (HepG2) and chronic myelogenous leukemia (K562), have particularly high numbers of expressed retrocopies [3]. We also identified three retrocopies, *AC107983.1*, *SYPL1P2*, and *NDUFB1P1*, whose expression occurred in all analyzed cancer libraries but not in normal tissues. In the case of two of them, *AC107983.1* and *NDUFB1P1*, genes localization and expression correlation suggest mechanisms of action based on *cis*-NAT regulation of the host genes *CCDC144B* and *CDC25A*, respectively. LncRNA *AC107983.1* also demonstrated microRNA sponging capability. This assumption was made based on shared miRNA targets and negative correlation of the expression [3].

Retro-lncRNAs associated with diseases described in this review, together with some additional published cases, are presented in Table 1.

## 3. Retrocopies as lncRNAs in Neurodegenerative Disorders

Neurodegenerative diseases constitute complex and heterogeneous conditions that are based on neurons devastating and mainly affect elderly people. This group, among others, involves Huntington’s disease (HD), Parkinson’s disease (PD) and Alzheimer’s disease (AD). Symptoms of these disorders are not clear-cut, and thus, the correct diagnosis is quite a challenge [87]. Our knowledge of neurodegenerative pathogenesis remains incomplete and to tackle these challenges, an increasing number of studies of neurodegenerative diseases take into account lncRNAs [88,89]. This is supported by the fact that some lncRNAs are exceptionally enriched in specific brain regions [90,91].

Costa et al. analyzed the differential expression of pseudogenes in neurodegenerative diseases. This analysis included retroposition-derived lncRNAs. In the case of Huntington’s disease, an interesting example is a group of three retrocopies, *HMGB1P1*, *HMGB1P5*, and *HMGB1P10*, which are related to disease phenotype and share microRNA binding sites with their progenitor [78]. Their parental gene, *HMGB1*, also plays a role in the neurodegeneration process. HMGB1 protein interacts directly with huntingtin protein, and the overexpression of *HMGB1* results in the inhibition of HD progression [92,93]. Complex analyses of RNA-seq data showed that one of *HMGB1* retrocopies, *HMGB1P10*, can act through microRNA sponging and may also regulate the *TPST2* gene in the *cis*-NAT configuration [3]. Another example of a dysregulated lncRNA is *TLK2P1* [78], which originates from the *TLK2* gene which has been connected with intellectual disabilities [94]. Our analysis suggested the action of *TLK2P1* as a microRNA sponge [3]. The expression level of the *VDAC1P1* retrocopy has also been deregulated in HD. On the basis of sharing binding sites with its parental gene, the role of the microRNA sponge can be inferred [78]. Although the involvement of its parental gene in HD was not reported, upregulation of the *VDAC1* gene was discovered in postmortem AD brains [95].

Explanations for the functionality of some HD-related retro-lncRNAs can be found in the role of their progenitors in brain development. Studies in mouse models have shown that *HMGN2*, the parental gene of *HMGN2P3*, is an important molecule involved in embryonic/postnatal brain development and that the loss of *HMGN2* is associated with microcephaly [96]. Another example of retrocopy with progenitors in brain formation is *FABP5P1*, where the *FABP5* gene participates in postnatal neurogenesis [97]. It has also been discovered that the expression of *HIGD1A*, a parental gene of *HIGD1AP14*, is widely distributed but uneven in the brain [98]. These two retro-lncRNAs, *FABP5P1* and *HIGD1AP14*, most likely constitute microRNA sponges, as they share common microRNA binding sites with their parental genes [78].

In the search for therapy, researchers of neurodegenerative diseases mainly focus on the use of induced pluripotent stem cells [99]. Costa et al. indicated that *POU5F1P4* is deregulated in HD [78]. The cognate gene *POU5F1* (*OCT4*) is one of the main pluripotent genes [100]. Furthermore, the parental gene of *RBBP4P4*, *RBBP4*, is also required for pluripotency maintenance [101]. Considering this fact, the occurrence of additional retrocopies of genes responsible for controlling this phenomenon may be essential to the outcome of therapy studies.

In the Parkinson’s disease dataset, also analyzed by Costa et al., three retroposition-derived lncRNAs, *PHC1P1*, *RBMXP2* and *CHCHD2P2*, were identified [78]. The parental gene of *PHC1P1*, *PHC1*, has been listed as involved in the neuroinflammation that underlies neurodegenerative diseases [102], and according to our analyses, its retrocopy may potentially compete for microRNAs [3]. The progenitor of *RBMXP2*, gene *RBMX*, is crucial during the development of the zebrafish brain [103] and has been related to X-linked intellectual disability [104]. The last retro-lncRNA, *CHCHD2P2*, showed an altered level of expression in HD as well as in PD. In the literature, there is a reference of its parental gene *CHCHD2* to the pathogenesis of Parkinson’s disease [105].

It is broadly discussed whether there is any relationship between neurodegenerative diseases and cancer. Both are age-related disorders, and they share the competitive endogenous RNA (ceRNA) mechanism involved in pathogenesis. In turn, these conditions differ in some contexts, as cancer is associated with cell death inhibition, while neurodegenerative diseases are connected with neuronal apoptosis [106]. These disorders share molecules dysregulated during disease progression, but sometimes changes in expression levels occur in opposite directions. It is worth noting that a negative correlation between the incidence of individual neoplasms and the risk of developing Alzheimer’s disease was reported [107]. Based on the results of Costa et al., who analyzed the differential expression of pseudogenes in neurodegenerative disease data [78], one may select retro-lncRNAs, which are common in both conditions. Examples of such are *POU5F1P4* and *PTENP1* as well as *RBMS1P1*, *RBMXP2* and *RHOQP2*, which were identified in our laboratory in the differential expression analysis of breast cancer RNA-seq data [108].

## 4. Retro-lncRNAs and Other Diseases

### 4.1. Cardiovascular Diseases

Cardiovascular diseases are described as disorders related to the heart and blood vessels, and they are the largest cause of all deaths worldwide [109]. This group of diseases includes peripheral arterial disease, coronary heart disease, rheumatic heart disease, congenital heart disease and others. These conditions include heart attack, stroke, essential hypertension (EH), aortic dissection and atrial fibrillation (AF). Although the most important risk factors are well known, we are still far from understanding the molecular basis of these diseases and finding effective treatments. Recently, lncRNAs have become one of the most promising targets in cardiovascular disease research.

*LOC646616* and *LAP3P2*, two retrogene-derived lncRNAs, have recently been described, together with two circRNAs, as candidates for therapeutic targets in the case of essential hypertension [79]. EH is defined as chronic high blood pressure without any definite cause, and it covers approximately 95% of all hypertension cases. *LOC646616* was identified as upregulated in EH patients and acted as a sponge for miR-637. As a result, the WNT/β-catenin signaling pathway is activated. This pathway is related to the regulation of stem cell pluripotency and cell fate during development. The second retro-lncRNA, *LAP3P2*, was highly coexpressed with mRNAs of crucial elements of the same WNT/β-catenin signaling pathway, *WNT* and *CAMK2N2* genes. It shares microRNA miR-637 binding sites with transcripts of these two genes and acts as competing endogenous RNA.

Another study related to cardiovascular diseases presented *VDAC2P2* as a lncRNA potentially involved in atrial fibrillation (AF). AF is one of the most common and complex types of arrhythmia and is related to the risk of stroke and heart failure. *VDAC2P2* together with two circRNAs were found to be significantly differentially expressed in atrial tissues in AF patients [80]. It was also shown that *VDAC2P2* may regulate its parental gene *VDAC2* as well as the neighboring gene *KLRG1*. Both parental gene and retrogene are related to the process of metabolite diffusion through the mitochondrial outer membrane. An association between mitochondrial dysfunction and atrial fibrillation has been previously postulated [110,111]. The function of *VDAC2P2* is not known, but it may be involved in homologous recombination and gene conversion or serve as an antisense RNA [80].

The differentiation, proliferation and apoptosis of smooth muscle cells (SMCs) are important factors related to the development of aortic dissection and aortic aneurysm (AA) [112]. LncRNAs are known to modulate this type of cell, and one of them is *PTENP1*. This is yet another molecular process in which this lncRNA is involved in addition to cancers and neurodegenerative disorders, as described above. *PTENP1* and its parental gene, *PTEN*, were found to be upregulated in human aortic dissection samples. In this case *PTENP1* also works as a microRNA sponge competing for miR-21 with parental gene transcripts, and overexpression of lncRNA results in elevated level of *PTEN* protein [81].

### 4.2. Mental Disorders 

Mental disorders are diverse diseases, but the most common characteristics include problems with perceptions, emotions, behavior and relationships with other people. This group includes depression, schizophrenia and other psychoses, dementia, bipolar disorder and autism spectrum disorders [109]. Recent studies have demonstrated that lncRNAs originating from retrocopies are also associated with this type of diseases. One of the examples is *NDUFV2P1*, which is related to schizophrenia (SZ), in which atypical neuronal transmission and dysregulation of brain energy metabolism were reported [113,114]. Last year, studies revealed a new basis of schizophrenia that is related to the abnormal functioning of mitochondria. The NDUFV2 protein, encoded by the *NDUFV2P1* progenitor, is a crucial subunit of the cytochrome C oxidase I (CoI) complex of the mitochondrial respiratory chain, and its level can be reduced due to increased retrocopy expression, which leads to mitochondrial dysfunction [82]. Interestingly, deterioration of the CoI complex was also observed in bipolar disorder and Parkinson’s disease [115,116].

Autism spectrum disorder (ASD) is diagnosed in one in 160 children worldwide and sometimes influences adolescents and adults. It is quite common to observe coexisting diseases, such as epilepsy, depression or attention deficit hyperactivity disorder (ADHD) [109]. The development of ASD is caused by multiple factors, among which there are more than 130 genes [83]. It has been reported that some lncRNAs may also be involved in ASD pathogenesis, especially at the epigenetic level [117]. One of them is *MSNP1AS*, an antisense transcript of *MSNP1*, a retrocopy that originated from the *MSN* gene. Downregulation of MSN protein expression leads to inhibited activation of the PI3K/Akt signaling pathway. *MNSP1AS* is one of the major factors influencing the PI3K/Akt pathway, and the RhoA and Rac1 pathways are important for neuronal structure and survival [83]. It was reported that *MSNP1AS* and *MSN* are able to form dsRNA (double stranded RNA), which suppresses the expression of the protein encoded by the parental gene [83,118,119]. Furthermore, overexpression of *MSNP1AS* in ASD patients has an impact on smaller amounts and lengths of neurites in human neural progenitor cell lines [118,119].

### 4.3. Other Diseases

Preeclampsia (PE) is a specific disease during pregnancy characterized by hypertension, sometimes accompanied by proteinuria. Severe preeclampsia (SPE) is a major cause of maternal death and perinatal mortality worldwide. Implantation, placentation and decidua formation are the key processes in early pregnancy, and disturbances are thought to be the major cause of PE [84]. Two interesting examples of retro-lncRNAs involved in PE are *HK2P1* [85] and *PGK1P2* [84]. *HK2P1* and its parental gene *HK2* were found to be downregulated in human endometrial stromal cells, which inhibits their proliferation and differentiation and causes preeclampsia. The retrocopy works as a ceRNA and regulates the expression of the parental gene through competition for miR-6887-3p [85]. A very similar mechanism of interaction was described for *PGK1P2* and *PGK1*. Deficiency of their mRNA levels and PGK1 protein in the decidua deregulates the glycolytic pathway, which is crucial for changes in the endometrium during pregnancy and the occurrence of PE. Additionally, retrocopy regulates the parental gene level by sponging microRNA, in this case miR-300-5p [84].

Biliary atresia (BA) is related to the fibrosis of extrahepatic bile ducts and is the major cause of cholestasis in children, which is the main reason for liver transplantation among children. The pathogenesis of biliary atresia is unclear, but some studies have shown an association between lncRNA deregulation and the development of fibrosis. One such lncRNA is *ANXA2P3*, which originates from a retrocopy of the annexin 2 gene *ANXA2*. Increased expression levels of *ANXA2P3* and *ANXA2* have a positive effect on cell proliferation and inhibit cell apoptosis. Transcripts of both genes are considered targets in treatments preventing liver injury and as future biomarkers in patients with BA [86].

Diabetes is a metabolic disease characterized by elevated levels of blood glucose resulting from the inefficient production or usage of endogenous insulin. It has a significant impact on human populations, as the number of people with diabetes has increased 4-fold over the last 40 years [109]. In some individuals affected by this disease, retrocopy-derived *HMGA1P8* lncRNA was shown to play an important role [69]. HMGA1 is an architectural nuclear protein that functions mainly as a specific cofactor for activation of the insulin receptor gene (*INSR*). Case studies of two unrelated patients affected by type 2 diabetes showed that the expression of the *HMGA1* gene was markedly reduced, while its retrocopy *HMGA1P8* was overexpressed. A more in-depth study indicated that enhanced expression of lncRNA results in destabilization of parental gene mRNA by effective competition for a *trans*-acting cytoplasmic protein critical to mRNA stability (Figure 2D). Consequently, the expression of the *INSR* gene is suppressed, which in turn results in insulin resistance [34].

## 5. Noncoding Retrocopies as Putative Players in Pathogenic Processes

**Figure 3:**
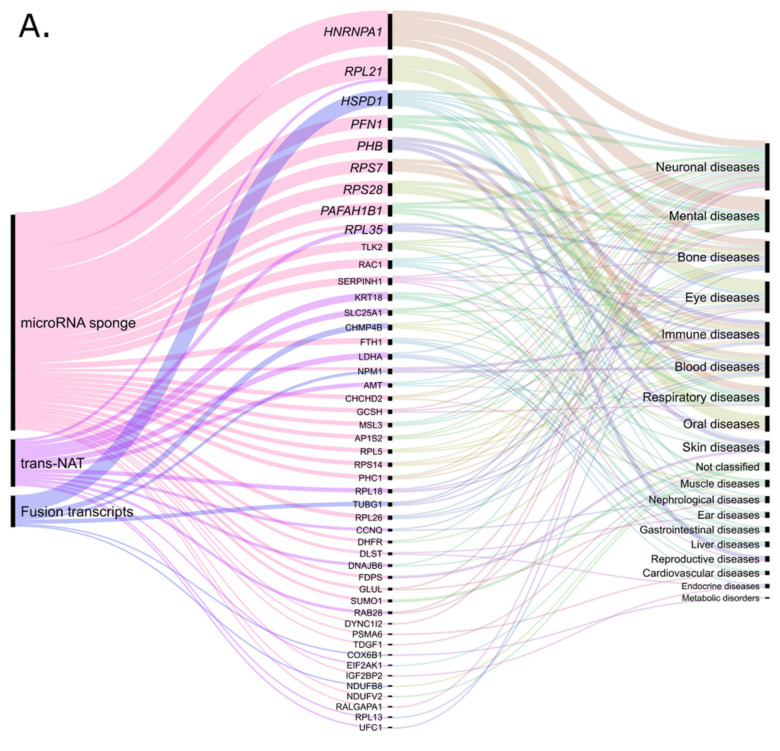

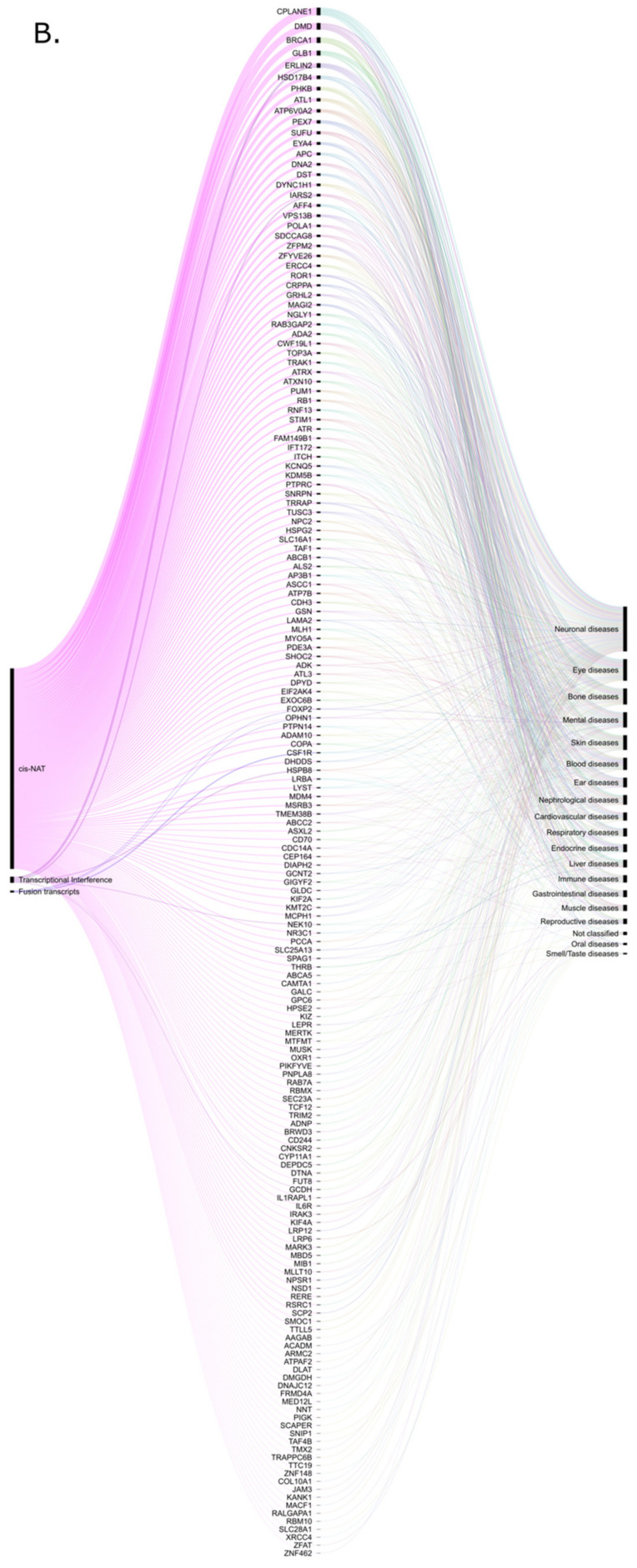
Classification of diseases associated with (**A**) parental genes and (**B**) host genes. (Plots created with RAWGraph [133])**.**

The numerous examples of the link between retrocopies and various pathogenic processes presented above are undoubtedly important evidence of their underestimated roles. These copies may be important for maintaining the proper functioning of the cell or, on the other hand, their expression may be deleterious. An increasing amount of data from a variety of high-throughput experiments makes it feasible to identify promising candidates that may regulate or interrupt the expression of other genes. In our recent work, we were able to propose the possible mode of action for approximately 43% of human retrocopies annotated in RetrogeneDB2 [3,27]. Utilizing these data we investigated whether this putatively regulated by retro-lncRNAs parental and host genes, are related to human diseases (Supplementary File S1). The input dataset included genes potentially regulated by retrocopies identified as (a) miRNA sponges; (b) trans natural antisense transcripts; (c) *cis* natural antisense transcripts; (d) factors of transcriptional interference and (e) source of fusion transcripts. These genes were selected based on genomic localization, RNA-seq data analysis, expression correlation analysis, identification of miRNA targets, sequence complementarity. All methodological aspects of these analyses are described in detail in original paper [3]. For each gene identified as putatively regulated by retrocopy, we retrieved the “MIM morbid accession” using Ensembl BioMart [120,121,122]. The resulting list of disease-related genes was then expanded by disease characteristics. To this goal, we classified the diseases according to the MalaCards global and anatomical categories [123]. Remarkably, both the parental and host genes are mainly associated with neuronal diseases. Bone, eye, and mental diseases were also well represented in the analyzed groups (Figure 3).

We found that 48 parental genes potentially regulated by 71 retrocopies were associated with 52 human diseases (Supplementary File S1: Table S1). In this group, microRNA sponge activity is the most popular mode of action (Figure 3A). This function was proposed for over three-quarters of the retrocopies. Among the identified parental genes, *CHCHD2* seems to be an interesting example. Recent studies suggest that *CHCHD2* regulates the functions of cytochrome c and that the loss of this regulation is associated with Parkinson’s disease [124]. We identified *retro_hsap_116*, also called *CHCHD2P6*, as a potential competitive endogenous RNA since it shares microRNA target sequences with the parental gene. Interestingly, *CHCHD2* may also be regulated in a similar way by another retrocopy, *CHCHD2P2* [78]. The predictions may be worth further analysis, especially considering the fact that recent studies showed specific changes in microRNA expression in Parkinson’s disease [125].

Retrocopies can also be a source of *trans* natural antisense transcripts for parental genes. From the analyzed dataset, 13 transcripts with retrocopy-derived exons in antisense orientation might regulate the expression of retrocopy progenitors. The retrocopy *retro_hsap_2353*, known as *KRT18P29*, embedded in the *PPP1R1C* gene can illustrate this phenomenon. Two noncoding isoforms of the host gene incorporated the 63 bp antisense sequence of *KRT18P29* retrocopy as a new exon. By definition, the exon is also antisense to *KRT18*, a parental gene that was found to be linked with liver cirrhosis. Interestingly, both parental and host genes were differentially expressed in the EGR1-overexpressing cell line used in a study on the malignancy of human non-small cell lung carcinoma [126]. However, when the expression of *KRT18* was upregulated, the expression of *PPP1R1C* was downregulated. In our previous studies, we found another intriguing example, an antisense transcript of retrocopy *AC021224.1-201*, which could be involved in splicing regulation of its progenitor *hnRNPA1* [29]. The analysis of RNA:RNA duplexes formed between lncRNAs and pre-mRNA sequences and predicted based on base-pairing analysis, suggested that this lncRNA is able to mask the 5′ splice site in the sixth intron of the parental gene (Figure 2B). When the interaction does not occur, a shorter isoform of *hnRNPA1* is expressed. This transcript was shown to play regulatory roles in human immunodeficiency virus splicing and replication [29,127].

Staying with the topic of natural antisense transcripts, it should be noted that the interaction of retrocopies in *cis* on host genes is the most represented group of their potential function (Figure 3B). In total, we found 186 retrocopies regulating 174 host genes associated with 247 diseases (Supplementary File S1: Table S2). A noteworthy subgroup is represented by intronic retrocopies proposed as transcriptional interference factors [3]. One example is the *ERLIN2* gene associated with spastic paraplegia type 18. The expression of embedded in this gene retrocopy (*retro_hsap_4044*) is positively correlated with the expression of two short splice variants of the host gene, which might suggest facilitating early transcription termination [3,36]. Moreover, *ERLIN2* was also studied in the context of other human diseases, such as mental retardation [128], lateral sclerosis [129] and breast cancer [130], which makes this gene worthy of further investigation.

In addition to the abovementioned functions, the nonallelic homologous recombination between a retrocopy and its progenitor, or two retrocopies of the same gene, may be considered as another important disease-related level of regulation. For example, a high sequence similarity between the *EIF2AK1* parental gene and *retro_hsap_2713* embedded in the *ATR* gene may explain the generation of a fusion transcript containing parental and host gene exons found in cancer cells [131]. In our data, nine retrocopies can be considered to be involved in the recombination of parental and/or host genes associated with diseases. We found, inter alia, a chimeric transcript associated with cataract and formed by *CHMP4B*, and *FBXO34*, the parental gene and host gene of *retro_hsap_1339*, respectively. Another example is a fusion of *OPHN1* related to the X-linked mental retardation host of *retro_hsap_4692,* and *AKIRIN1* (Figure 2E), the retrogene progenitor. Moreover, retrocopies themselves can be a part of chimeric transcripts. An example could be two chimeric transcripts found in acute myeloid leukemia. One resulted from the fusion of *retro_hsap_1547* and its parental gene *RPL32*, and another resulted from *retro_hsap_4032* and the parental gene *COX6B1* [3,132].

## 6. Conclusions

In this review, we described multiple examples of retrocopy-derived lncRNAs related to human diseases. These examples demonstrate how important players in the whole cell machinery are copies commonly described as pseudogenes. They work in various manners, including microRNA sponging, chimeric transcript formation, as NATs or by influencing splicing. Their functions have been associated with many disorders, such as cancer, neurodegenerative and mental diseases, and cardiovascular diseases. Involvement of retrocopies in those pathogeneses, for a long time underrated, has proven to be crucial and confirms the role of retrocopies in many molecular mechanisms. Numerous studies have also demonstrated their potential as therapeutic targets. Apart from the literature-based examples, we report here additional candidates selected based on our bioinformatics analysis, that may regulate their parental and host genes. This gives many promising candidates for further studies and helps to understand retroposed gene involvement in human diseases.

## Figures and Tables

**Table 1:** Retro-long noncoding RNAs (lncRNAs) in human diseases.

Retro-lncRNA	Verified or Putative Mechanism	Diseases	Parental Gene
Cancer			
*HMGA1P6*	miRNA sponge [43]	endometrial cancer [42], ovarian cancer, thyroid cancer [43]	*HMGA1*
*HMGA1P7*	miRNA sponge [43]		*HMGA1*
*KRASP1*	miRNA sponge [15]	prostate cancer [15]	*KRAS*
*POU5F1P4 (Oct4-pg4)*	miRNA sponge [48]	hepatocellular carcinoma [48]	*POU5F1 (OCT4)*
*POU5F1P5 (Oct4-pg5)*	miRNA sponge [49], epigenetic regulation [50]	endometrial carcinoma [49]	*POU5F1 (OCT4)*
*SUMO1P3*	miRNA sponge [3,65]; *cis*-NAT for host gene [3]	gastric cancer [52], hepatocellular carcinoma [65]	*SUMO1*
*RACGAP1P*	miRNA sponge [54,55]	hepatocellular carcinoma [54], breast cancer [55]	*RACGAP1*
*ANXA2P2*	-	hepatocellular carcinoma [59]	*ANXA2*
*UBE2CP3*	-	hepatocellular carcinoma [60]	*UBE2CP3*
*INTS6P1*	miRNA sponge [61]	hepatocellular carcinoma [61]	*INTS6*
*PTENP1*	miRNA sponge [66], epigenetic regulation [30]	hepatocellular carcinoma [66], glioma [62]	*PTEN*
*TUSC2P1*	miRNA sponge [64]	esophageal squamous cell carcinoma [67]	*TUSC2*
*PDIA3P1*	miRNA sponge, *cis*-NAT for host gene [3]	hepatocellular carcinoma [68]	*PDIA3*
*CSDAP1 (YBX3P1)*	-	lung cancer [69]	*CSDA (YBX3)*
*LGMNP1*	-	glioblastoma [70]	*LGMN*
*PTTG3P*	miRNA sponge [71]	breast cancer [71]	*PTTG1*
*CKS1BP7*	-	breast cancer [72]	*CKS1B*
*MSL3P1*	miRNA sponge [3]	renal cell carcinoma [73]	*MSL3*
*CTNNA1P1*	miRNA sponge [74]	colorectal cancer [74]	*CTNNA1*
*PPIAP43*	miRNA sponge [75]	lung cancer [75]	*PPIA*
*FTH1P3*	miRNA sponge [76]	breast cancer [76]	*FTH1*
*E2F3P1*	-	hepatocellular carcinoma [77]	*E2F3*
*AC107983.1*	miRNA sponge, *cis*-NATs for host gene [3]	cancer cell lines [3]	*RPS28*
*SYPL1P2*	-		*SYPL1*
*NDUFB1P1*	*cis*-NATs for host gene [3]		*NDUFB1*
**Neurodegenerative Disorders**			
*BZW1P2*	miRNA sponge [78]	Huntington’s disease [78]	*BZW1*
*COX7A2P2*	miRNA sponge [78]		*COX7A2*
*DGKZP1*	miRNA sponge [78]		*DGKZ*
*EEF1A1P5*	miRNA sponge [78], *cis*-NAT for host gene [3]		*EEF1A1*
*EIF2S2P4*	miRNA sponge [78], fusion transcript [3]		*EIF2S2*
*ETF1P1*	miRNA sponge [78]		*ETF1*
*FABP5P1*	miRNA sponge [78]		*FABP5*
*HIGD1AP14*	miRNA sponge [78]		*HIGD1A*
*HMGB1P1*	miRNA sponge [78]		*HMGB1*
*HMGB1P10*	miRNA sponge [3,78]; *cis*-NAT for host gene [3]		*HMGB1*
*HMGB1P5*	miRNA sponge [78]		*HMGB1*
*HMGN1P36*	miRNA sponge [78]		*HMGN1*
*HMGN2P3*	miRNA sponge [78]		*HMGN2*
*HNRNPA3P1*	miRNA sponge [78]		*HNRNPA3*
*HTR7P1*	miRNA sponge [78]		*HTR7*
*POU5F1P4 (Oct4-pg4)*	miRNA sponge [78]		*POU5F1(OCT4)*
*PTENP1*	miRNA sponge [78]		*PTEN*
*RBBP4P4*	miRNA sponge [78]		*RBBP4*
*RBMS1P1*	miRNA sponge [3,78], *cis*-NAT for host gene [3]		*RBMS1*
*RHOQP2*	miRNA sponge [78]		*RHOQ*
*RPLP0P6*	miRNA sponge [78]		*RPLP0*
*S100A11P1*	miRNA sponge [78]		*S100A11*
*SKP1P1*	miRNA sponge [78]		*SKP1*
*TLK2P1*	miRNA sponge [3,78]		*TLK2*
*VDAC1P1*	miRNA sponge [78]		*VDAC1*
*VEZF1P1*	miRNA sponge [78]		*VEZF1*
*YWHAZP3*	miRNA sponge [78]		*YWHAZ*
*ZFAND6P1*	miRNA sponge [78]		*ZFAND6*
*CHCHD2P2*	miRNA sponge [78]	Parkinson’s disease [78]	*CHCHD2*
*PHC1P1*	miRNA sponge [3,78]		*PHC1*
*RBMXP2*	miRNA sponge [78]		*RBMX*
*CHCHD2P2*	miRNA sponge [78]	Huntington’s disease, Parkinson’s disease [78]	*CHCHD2*
**Other Diseses**			
*LOC646616*	miRNA sponge [79]	essential hypertension [79]	*TMEM183A*
*LAP3P2*			*LAP3*
*VDAC2P2*	-	atrial fibrillation [80]	*VDAC2*
*PTENP1*	miRNA sponge [81]	aortic dissection [81]	*PTEN*
*NDUFV2P1*	miRNA sponge [3]	shizophrenia [82]	*NDUFV2*
*MSNP1AS*	-	autism spectrum disorder [83]	*MSN*
*PGK1P2*	miRNA sponge [84]	(severe) preeclampsia [84]	*PGK1*
*HK2P1*	miRNA sponge [85]	(severe) preeclampsia [85]	*HK2*
*ANXA2P3*	-	biliary atresia [86]	*ANXA2*
*HMGA1P8*	competition for factor [34]	diabetes [34]	*HMGA1*

## Supplementary Materials

The following are available online at https://www.mdpi.com/article/10.3390/cells10040912/s1, Table S_Table_1, S_Table_2; Supplementary_file_1.xlsx.
